# Micron-Sized Thiol-Functional Polysilsesquioxane Microspheres with Open and Interconnected Macropores: Effects of the System Composition on the Porous Structure and Particle Size of the Microspheres

**DOI:** 10.3390/molecules29122841

**Published:** 2024-06-14

**Authors:** Lu Han, Zhenyu Nie, Rongsheng Gao, Chengyou Kan

**Affiliations:** Key Laboratory of Advanced Materials of Ministry of Education, Department of Chemical Engineering, Tsinghua University, Beijing 100084, China

**Keywords:** polysilsesquioxane, micron-sized microsphere, macroporous, thiol, control

## Abstract

Control of the porous structure and particle size is essential for improving the properties of polysilsesquioxane (PSQ) microspheres. Herein, using the strategy combining inverse suspension polymerization, two-step sol–gel- and polymerization-induced phase separation processes, micron-sized thiol-containing macroporous PSQ (TMPSQ) microspheres with controllable morphologies, adjustable particle diameters (4.9–17.3 μm), and pore sizes (40–3774 nm) were prepared. The morphology and size of the TMPSQ microspheres were characterized by SEM. The mercury intrusion method was employed to analyze the porous structure of the microspheres. The effects of the composition of the sol–gel disperse phase, the mass ratio of the sol–gel disperse phase to the oil continuous phase (WR_W/O_), and the Span 80 mass content in the oil continuous phase on the morphology, particle diameter and pore size of the TMPSQ microspheres were investigated. Results indicated that the composition of the sol–gel disperse phase determines the morphology and porous structure of the microspheres, and WR_W/O_ and Span 80 content have remarkable impacts on the morphology and particle size of the microspheres. This study is beneficial to the design and fabrication of functional PSQ microspheres with desired properties and promising application prospects.

## 1. Introduction

Owing to the inorganic –Si–O–Si– framework and variable organic groups, polysilsesquioxane (PSQ) microspheres have unique and beneficial features, such as great mechanical performance, stability, biocompatibility, temperature resistance, and easy functionalization [1]. Therefore, PSQ microspheres are ideal materials for light-scattering agents [2], pollutant adsorbents [3], chromatographic stationary phases [4], catalyst supports [1], and biosensors [5]. To satisfy the needs for diverse applications, controlling particle size and size distribution has become the focus in the area of PSQ microspheres [6,7,8].

In the past few decades, porous materials have attracted considerable interest from industries and researchers. Due to their advantages of large surface area, low density, and unique structure, porous materials have better performance than nonporous materials in many fields. Until now, researchers have always endeavored to obtain porous materials with tunable porous structures to improve their properties and meet different requirements, which depend on the desired speed of mass transfer or release of substances with target molecular sizes [9,10,11,12]. Macropores, with the pore size over 50 nm, can facilitate mass transfer and enhance interactions between the active sites on the surface and the substances outside the materials [13]. Additionally, the movement of large molecules along the macropore channels is allowed, which expands the application potential of porous materials [14]. Yet introducing macropores into various functional materials and adjusting the pore sizes are still challenging.

**Figure 1:**
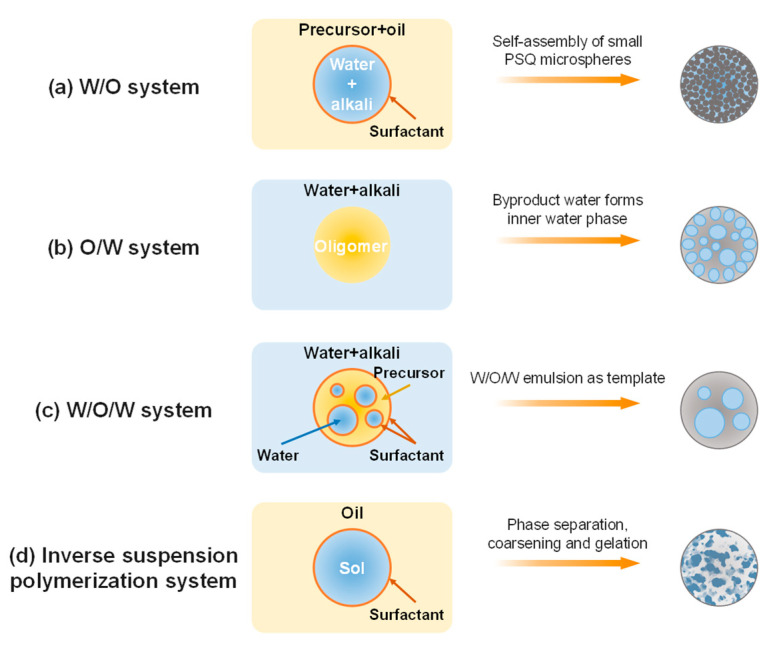
Schematic illustration of preparing porous PSQ microspheres in various emulsion or suspension systems. (**a**) W/O system [22], (**b**) O/W system [23,24], (**c**) W/O/W system [1], and (**d**) inverse suspension polymerization system [25].

The functionality of PSQ microspheres can be enhanced by porous structures, but there is little research on porous PSQ microspheres compared to porous silica-based microspheres with a similar –Si–O–Si– skeleton. Multiple methods have been employed to fabricate porous silica-based microspheres, such as template [15,16], colloid aggregation [17], self-assembly [18], spray drying [19], aerosol [20], and polymerization-induced phase separation [13,21] methods. However, the synthesis of macroporous PSQ microspheres generally resorts to emulsion or suspension systems—such as W/O [22], O/W [23,24], and W/O/W [1] systems—as illustrated in Figure 1 because of their mild synthetic conditions and moderate post-treatment processes. In our previous study, a new strategy combining inverse suspension polymerization, two-step sol–gel and polymerization-induced phase separation processes was developed to synthesize thiol-containing open macroporous polysilsesquioxane (TMPSQ) microspheres, and the formation mechanism of the bicontinuous structure was revealed through the following processes of spinodal decomposition, coarsening, gelation, and the removal of solvent [25]. Tuning the pore size and pore structure of TMPSQ microspheres can control the surface area, active sites on the surface, and the mass transfer rate. The design and preparation of TMPSQ microspheres with different pore sizes and morphologies are beneficial to broaden their application fields. Nevertheless, control synthesis of TMPSQ microspheres with tunable pore sizes has not been achieved yet.

In this study, the TMPSQ microspheres with controllable morphology, particle diameter, and pore size were prepared via the same strategy as above using the mixture of H_2_O, MeOH, methyltrimethoxysilane (MTMS), (3-mercaptopropyl)trimethoxysilane (MPTMS) and catalyst solutions as the sol–gel disperse phase and liquid paraffin and Span 80 as the oil continuous phase. The effects of the composition of the polymerization system on the morphology, particle diameter, and pore size of the microspheres were systematically investigated.

## 2. Results and Discussion

### 2.1. Effects of the Composition of the Sol–Gel Disperse Phase

#### 2.1.1. Effect of the Molar Ratio of H_2_O to Total Precursors (MR_H2O/Si_)

The siloxane skeletons and organic side groups of TMPSQ microspheres were confirmed by FTIR, Raman spectroscopy (Figure S1a,b) [25] and the solid-state ^29^Si NMR (Figure S2), and details were given in Supplementary Information.

In the labels of all samples in this article, the alphabetical order is the order of the factor being investigated; the numerical order reflects the value of the respective factor, and the higher the order, the higher the value.

**Figure 2:**
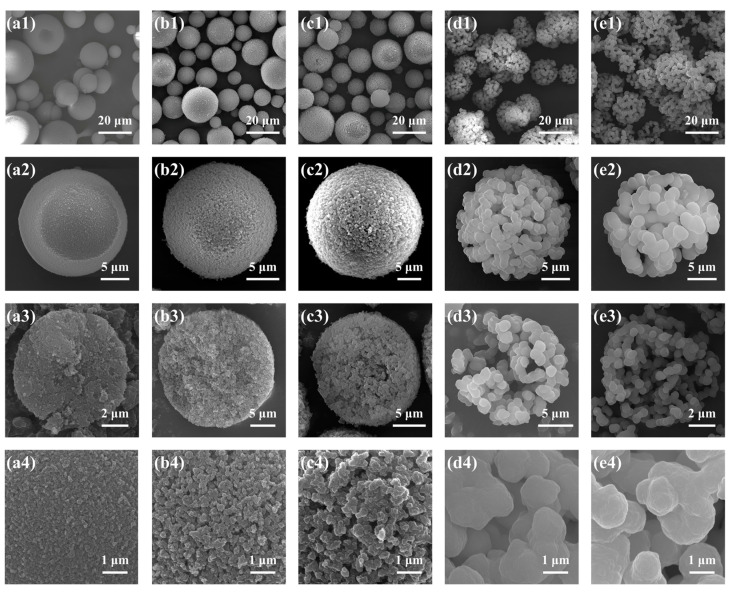
SEM photographs of the TMPSQ microspheres prepared with different MR_H2O/Si_ at different magnifications: (**a1**–**a4**) 7:1 (sample A1), (**b1**–**b4**) 8:1 (sample A2), (**c1**–**c4**) 8.5:1 (sample A3), (**d1**–**d4**) 9:1 (sample A4), and (**e1**–**e4**) 10:1 (sample A5).

**Figure 3:**
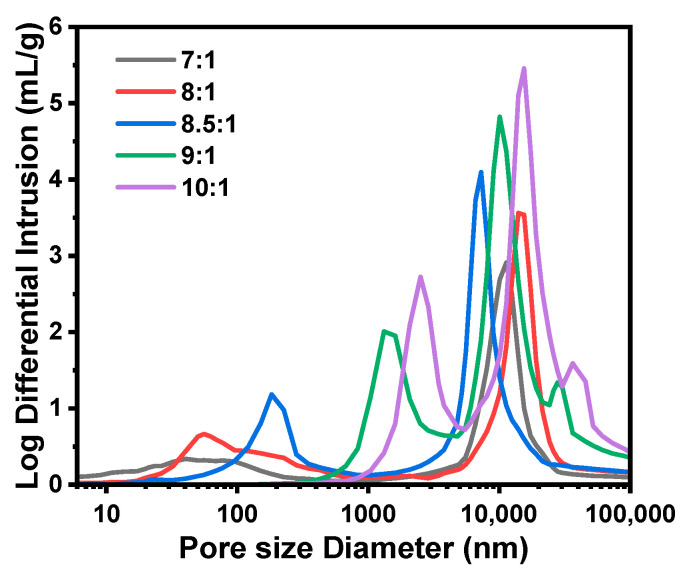
Pore size distributions of the TMPSQ microspheres prepared with different MR_H2O/Si_.

Samples A1–A5 were prepared with the increase in MR_H2O/Si_ from 7:1 to 10:1. Effect of the MR_H2O/Si_ in the sol–gel disperse phase was investigated by varying Water-A and Water-B, and the recipe and properties of TMPSQ microspheres are listed in Table 1. Here, the water amount of the mixture of 0.01 M HCl (aq) and Water-A was equal to that of the mixture of NH_4_OH (aq, 10 wt.%) and Water-B. As presented in Figure 2, all samples had open porous structures. In this study, pore characteristics of the TMPSQ microspheres were determined using the mercury intrusion method, and the log differential intrusion versus pore size curves were used to assess the pore size distributions. According to Figure 3, all samples were of bimodal distributions, indicating the coexistence of intraparticle pores and interparticle voids. When the MR_H2O/Si_ was 7:1 (sample A1), *D*_intra_ was only 40 nm, suggesting that the pores were mesopores rather than macropores. With the increase in MR_H2O/Si_, *V*_pore_, *P*_total_, *D*_intra_, *V*_intra_, and *P*_total_ all increased, while *S*_MIP_ decreased, which suggested that the pore in the microspheres became larger. Under lower MR_H2O/Si_ (from 7:1 to 8.5:1), the particles had higher sphericity (samples A1–A3). When this ratio reached 9:1 (sample A4), some microspheres were slightly damaged. With a higher MR_H2O/Si_ at 10:1 (sample A5), the sphericity of particles was poorer, and some non-spherical fragments were observed in Figure 2(e1), indicating that the microspheres were partly broken during preparation.

It has been demonstrated that H_2_O content has a great impact on phase separation in the sol–gel process of alkoxysilanes [26]. With more water in the disperse phase, the polarity of the solvent becomes higher, which causes lower compatibility between the oligomers and the solvent and results in a stronger phase separation in the disperse phase. Thus, with higher MR_H2O/Si_, the oligomer and solvent phases caused by spinodal decomposition grew fully and both the skeletons and pores in microspheres became larger. However, when the MR_H2O/Si_ was over 9:1, the coarsening process became remarkable and the fragmentation of the oligomer phase started [26,27]. In this case, the skeletons of microspheres became weakly connected and were partly broken by washing and drying. For the sample A5 with the highest MR_H2O/Si_ of 10:1, the more significant coarsening process caused the further breakup of the oligomer phase. Hence, more broken microspheres and fragments were observed in Figure 2(e1). Even though microspheres with similar structures as sample A5 had larger pores, their disadvantages would limit their application potential. On the one hand, their specific surface areas became lower, and as a result, some properties—such as loading and adsorption capacities—would be weakened. On the other hand, this type of microsphere was more fragile and easily broken into smaller particles and fragments, which made it more difficult to recover and reuse the microsphere.

#### 2.1.2. Effect of the Molar Ratio of MeOH to Total Precursors (MR_MeOH/Si_)

**Figure 4:**
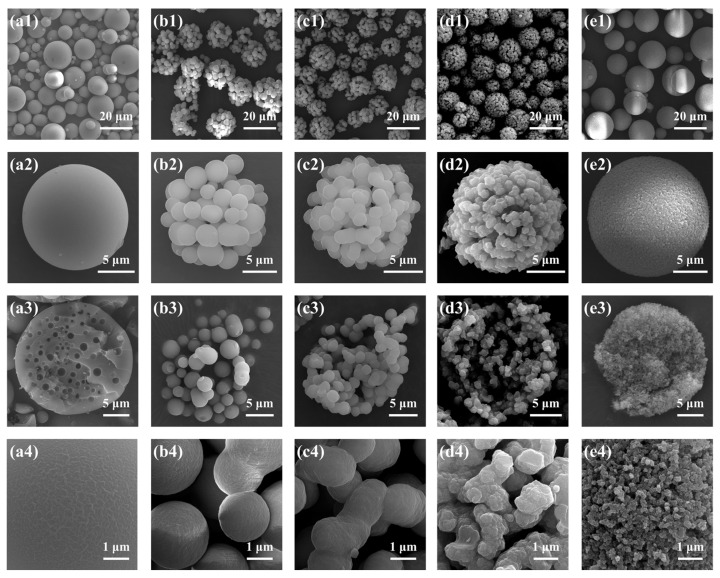
SEM photographs of the TMPSQ microspheres prepared with different MR_MeOH/Si_ at different magnifications: (**a1**–**a4**) 0:1 (sample B1), (**b1**–**b4**) 1:1 (sample B2), (**c1**–**c4**) 2:1 (sample B3), (**d1**–**d4**) 3:1 (sample B4), and (**e1**–**e4**) 5:1 (sample B6).

**Figure 5:**
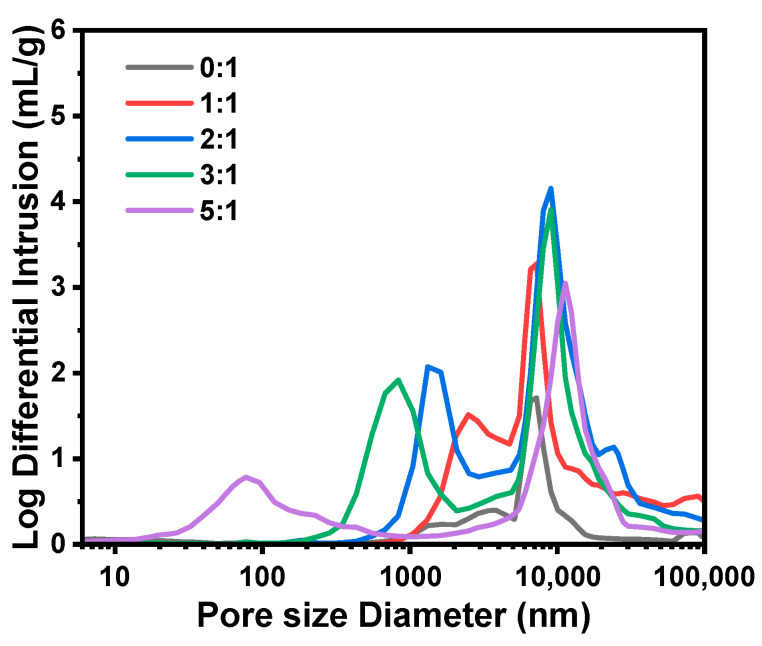
Pore size distributions of the TMPSQ microspheres prepared with different MR_MeOH/Si_.

Samples B1–B6, with gradually increasing MR_MeOH/Si_ from 0:1 to 5:1, were synthesized with varying amounts of MeOH, and the results are presented in Table 2 and Figure 4 and Figure 5, respectively. In comparison with other samples, sample B1 (MR_MeOH/Si_ = 0:1) was rather special. The surface was smooth, and no open pore was found (Figure 4(a4)), and only one main peak attributed to the interparticle spaces existed in Figure 5, indicating the absence of open macropores in the microspheres. Nevertheless, closed pores with an average diameter of 1.06 ± 0.30 μm were formed inside the microspheres (Figure 4(a3)). The reason is that when no MeOH was added, the composition of the sol–gel disperse phase was off-critical and located in the metastable region in the equilibrium phase diagram [27], and the phase separation in sample B1 conformed to the nucleation-growth mode, forming the isolated multiple cavities inside microspheres [28]. For the samples from B2 to B6, open porous structures and bimodal pore size distributions were observed. With the increase in MR_MeOH/Si_ from 1:1 to 5:1, *D*_intra_ decreased and *S*_MIP_ increased dramatically, suggesting a reduction in pore size. The phase separation was suppressed to generate finer bicontinuous structures inside the microspheres with higher MeOH content, which is consistent with the previous studies [26,29]. Despite the macroporous structure, the morphology of sample B2 (MR_MeOH/Si_ = 1:1) was still different from that of samples B3–B6 (with the MR_MeOH/Si_ from 2:1 to 5:1). The skeletons of sample B2 consisted of numerous isolated or weakly connected smaller microspheres (Figure 4(b4)), but the bicontinuous structures were generated in samples B3–B6 (Figure 2(c4) and Figure 4(c4–e4)). With the MR_MeOH/Si_ of 1:1 (sample B2), the composition was also off-critical but still in the unstable region in the phase diagram, and the coarsening process was rather remarkable, which caused the fragmentation and spheroidization of the oligomer domain and resulted in the aggregation of smaller spherical particles [30]. Additionally, this weak connection between spherical units was partly ruined during washing and drying, and some fragments could be seen in the SEM photograph (Figure 4(b1)).

#### 2.1.3. Effect of the Molar Ratio of NH_4_OH to Total Precursors (MR_NH4OH/Si_)

**Figure 6:**
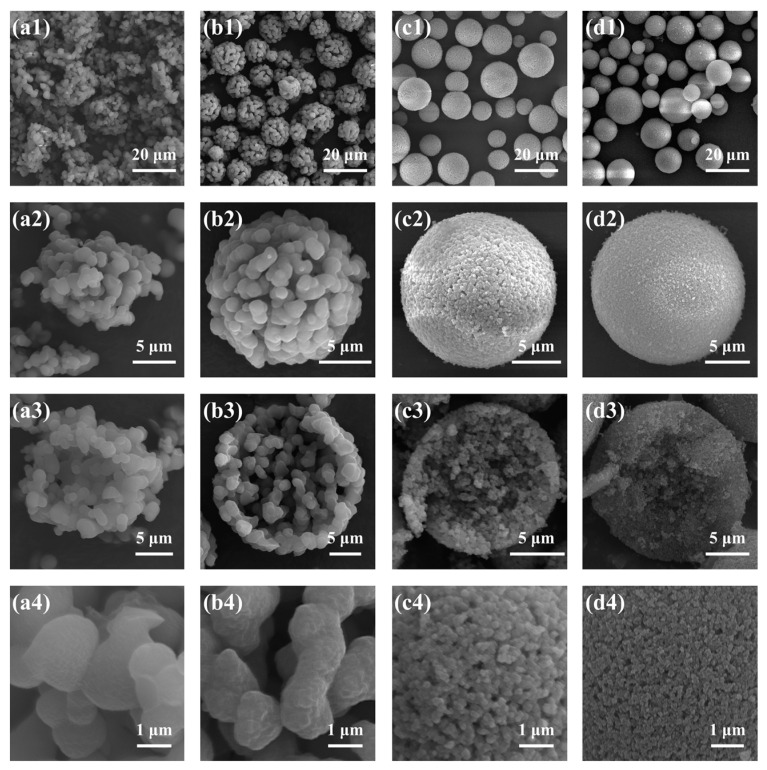
SEM photographs of the TMPSQ microspheres prepared with different MR_NH4OH/Si_ at different magnifications: (**a1**–**a4**) 4.7 × 10^−3^:1 (sample C1), (**b1**–**b4**) 7.0 × 10^−3^:1 (sample C2), (**c1**–**c4**) 11.7 × 10^−3^:1 (sample C4), and (**d1**–**d4**) 14.1 × 10^−3^:1 (sample C5).

**Figure 7:**
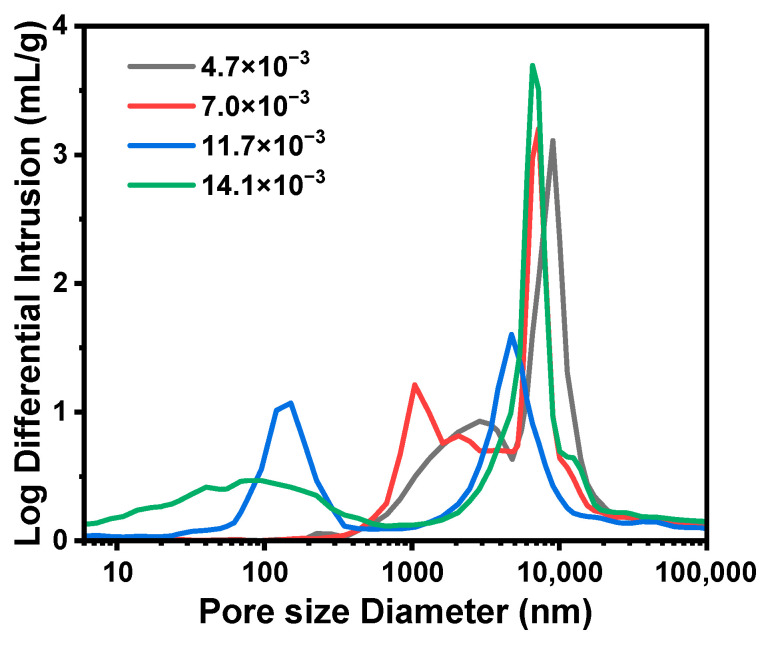
Pore size distributions of the TMPSQ microspheres prepared with different MR_NH4OH/Si_.

Table 3 summarizes the results of TMPSQ microspheres (samples C1–C5) prepared with the increase in MR_NH4OH/Si_ from 4.7 × 10^−3^:1 to 14.1 × 10^−3^:1. In preparation, with the same MR_H2O/Si_ at 8.5:1, the dosages of NH_4_OH (aq, 10 wt.%) and Water-B were varied to change the MR_NH4OH/Si_. The morphologies and pore size distributions of the TMPSQ microspheres are presented in Figure 6 and Figure 7. Results showed that with the increase in MR_NH4OH/Si_, *D*_intra_, *V*_intra_, and *P*_total_ decreased, and *S*_MIP_ increased. All the samples had open pores and bimodal pore size distributions, and both the domains of skeletons and pores became narrower with higher MR_NH4OH/Si_. Note that *D*_mean_ of sample C1 (MR_NH4OH/Si_ = 4.7 × 10^−3^:1) was lower than that of the others, and there were numerous small particles (Figure 6(a1)), which came from the broken microspheres and fragments of the skeletons.

In this series of TMPSQ microspheres, since MR_H2O/Si_ and MR_MeOH/Si_ remained constant, the compatibility between the solvent and oligomers was considered to be unchanged, and the change in MR_NH4OH/Si_ did not significantly affect the phase separation. However, the increase in MR_NH4OH/Si_ can greatly accelerate the condensation rate and reduce the gelation time. With a higher MR_NH4OH/Si_, the interconnected structure was frozen in earlier stages of spinodal decomposition, and the coarsening process was inhibited. In this case, both the oligomer phase and the solvent phase became finer, resulting in smaller pore sizes inside the microspheres.

#### 2.1.4. Effect of the MPTMS/MTMS Molar Ratio (MR_MPTMS/MTMS_)

**Figure 8:**
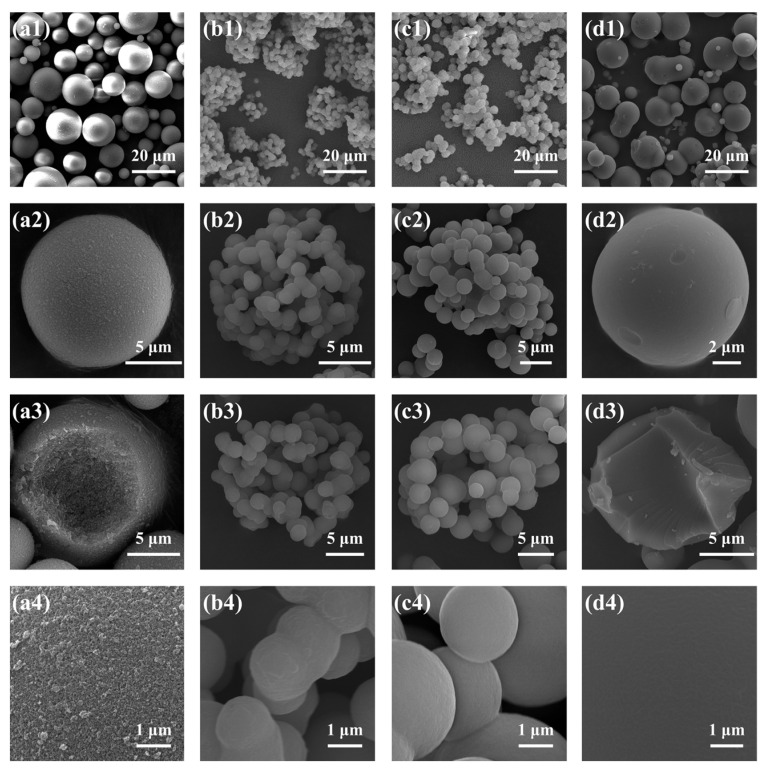
SEM photographs of the TMPSQ microspheres prepared with different MR_MPTMS/MTMS_ at different magnifications: (**a1**–**a4**) 0:1 (sample D1), (**b1**–**b4**) 1:4 (sample D3), (**c1**–**c4**) 1:3 (sample D4), and (**d1**–**d4**) 1:2 (sample D5).

**Figure 9:**
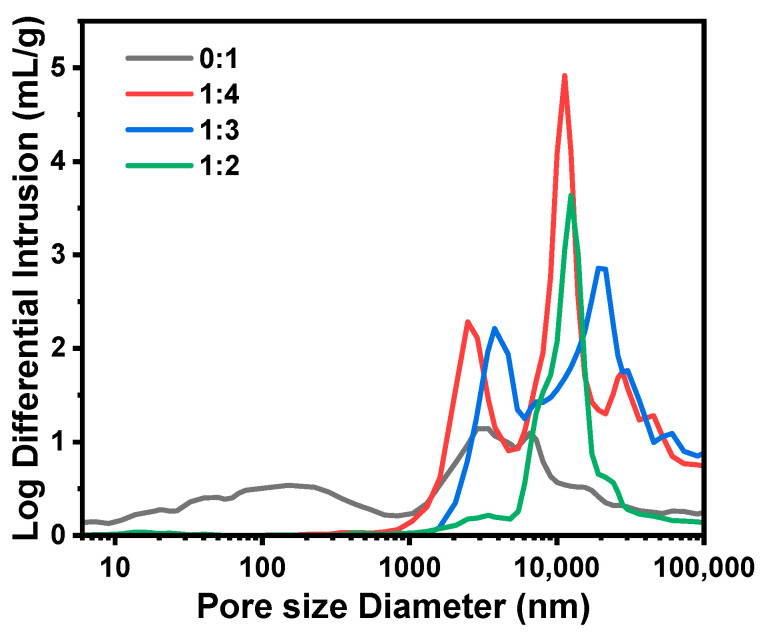
Pore size distributions of the TMPSQ microspheres prepared with different MR_MPTMS/MTMS_.

The hydrophobicity of the precursors strongly affects the phase separation in the sol–gel system [26]. Here, MPTMS has a longer carbon chain in the side groups and is thus more hydrophobic than MTMS. In order to investigate the effect of MR_MPTMS/MTMS_, Samples D1–D5 were prepared with MR_MPTMS/MTMS_ of 0:1, 1:5, 1:4, 1:3, 1:2, and 1:1, respectively, but the same 0.06 mol of total precursors, and the results are summarized in Table 4, Figure 8 and Figure 9. 

The microspheres prepared with only MTMS (sample D1) were complete spheres (Figure 8(a1–a4)) with the lowest *D*_intra_ (151 nm) and the highest *S*_MIP_ (70.8 m^2^/g) among samples D1–D5. Due to the less hydrophobic methyl, MTMS-derived oligomers were more compatible with the solvent than MTMS–MPTMS-derived oligomers, and the phase separation was less remarkable. Thus, the skeletons and pores of the microspheres became finer. When the MR_MPTMS/MTMS_ increased to 1:4 (sample D3), *D*_intra_ drastically increased to 2485 nm, *S*_MIP_ decreased to 2.1 m^2^/g, and the microspheres started to be broken and non-spherical (Figure 8(b1)). Obviously, the phase separation was quite sensitive to MPTMS content, and a small amount of MPTMS could cause a noticeable coarsening process. When the MR_MPTMS/MTMS_ reached 1:3 (sample D4), *D*_intra_ further increased to 3774 nm, *S*_MIP_ decreased to 1.3 m^2^/g, and the skeletons were made up of weakly connected small particles (Figure 8(c1–c4)), meaning that fragmentation and spheroidization occurred during the coarsening process. Since the structure was relatively loose, the microspheres were easily broken during washing and drying. Surprisingly, when the MR_MPTMS/MTMS_ reached 1:2 (sample D5), the solid microspheres with the smallest *D*_mean_ (8.3 μm) were obtained. As shown in Figure 8(d1–d4), there existed no obvious macroporous structure, and only a main peak was observed in Figure 9. With an intense phase separation at a higher MPTMS content, the coarsening process proceeded quickly and thoroughly, and the sol was separated into two totally dissociative phases [27], and the isolated oligomer droplets were eventually converted into the solid microspheres.

### 2.2. Effect of the Mass Ratio of Sol–Gel Disperse Phase to Oil Continuous Phase (WR_W/O_)

**Figure 10:**
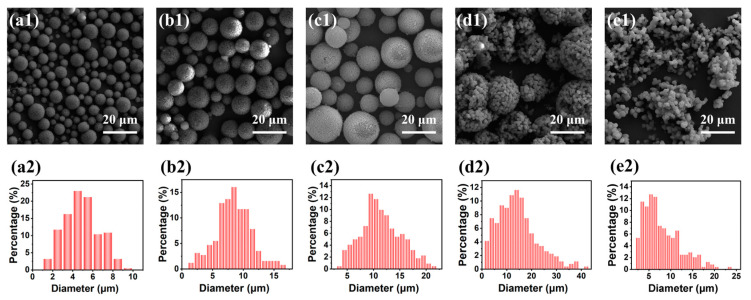
(**a1**–**e1**) SEM photographs and (**a2**–**e2**) particle size histograms of the TMPSQ microspheres prepared with different WR_W/O_: (**a1**,**a2**) 0.11:1 (sample E1), (**b1**,**b2**) 0.22:1 (sample E2), (**c1**,**c2**) 0.32:1 (sample E3), (**d1**,**d2**) 0.43:1 (sample E4), and (**e1**,**e2**) 0.54:1 (sample E5).

A series of TMPSQ microspheres (samples E1–E5) were synthesized by varying WR_W/O_ from 0.11:1 to 0.54:1 with the same compositions of sol–gel disperse phase and oil continuous phase, and results were presented in Table 5 and Figure 10. For the samples E1–E3 (with the WR_W/O_ from 0.11:1 to 0.32:1), the microspheres had great sphericity and integrity, and the interconnected macroporous structures were observed. Additionally, their size distributions were unimodal (Figure 10(a2–c2)), suggesting that the inverse suspension polymerization process was stable enough, and the integrity of droplets and the resultant microspheres was preserved. With the increase in the WR_W/O_, *D*_mean_ increased from 4.9 to 11.4 μm, but CV and *D*_intra_ did not change remarkably, which indicated that adjusting the WR_W/O_ is an effective method of tuning particle size.

However, when the WR_W/O_ increased to 0.43:1 (sample E4), *D*_mean_ reached the maximum (14.4 μm), but CV and *D*_intra_ dramatically increased to 56.0% and 1606 nm, respectively, and partially broken large microspheres and small fragments were observed in Figure 10(d1). Further increasing WR_W/O_ to 0.54:1 (sample E5), the value of *D*_mean_ sharply reduced to 7.9 μm, and the products were non-spherical and fragmented (Figure 10(e1)). In the case of a higher WR_W/O_, larger sol–gel disperse phase droplets were generated, and the polymerization system became unstable. During polymerization process, the breakup and coalescence of droplets co-existed, and the loose structure instantly formed inside the droplets could be easily destroyed by these two dynamic processes, resulting in non-spherical products.

### 2.3. Effect of the Span 80 Mass Content in the Oil Continuous Phase

**Figure 11:**
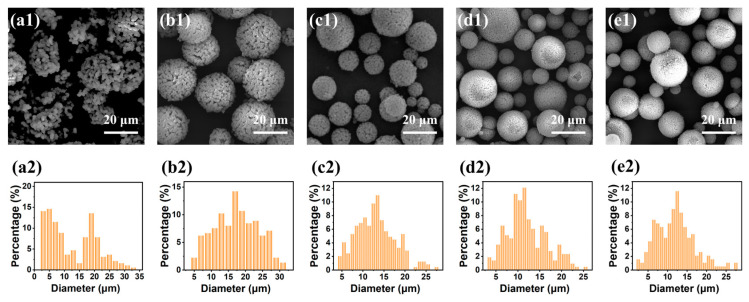
(**a1**–**e1**) SEM photographs and (**a2**–**e2**) particle size histograms of the TMPSQ microspheres prepared with different Span 80 mass contents in the oil continuous: (**a1**,**a2**) 1 wt.% (sample F1), (**b1**,**b2**) 2 wt.% (sample F2), (**c1**,**c2**) 4 wt.% (sample F3), (**d1**,**d2**) 8 wt.% (sample F5), and (**e1**,**e2**) 10 wt.% (sample F6).

As a stabilizer, adequate Span 80 dosage was a key factor in controlling the size and integrity of the dispersed phase droplets. A series of TMPSQ microspheres (samples F1–F6) was prepared with increasing Span 80 mass contents in the oil phase from 1 wt.% to 10 wt.%, and the results are given in Table 6 and Figure 11. When the Span 80 content was as low as 1 wt.% (sample F1), the obtained microspheres were cracked and non-spherical (Figure 11(a1)), the size distribution was bimodal (Figure 11(a2)), and the peaks were at around 5 μm and 19 μm, respectively. This indicated that the amount of Span 80 was not enough to stabilize the droplets against breakup and coalescence and that the droplets were easily destroyed by stirring. For samples F2–F4 with Span 80 contents from 2 wt.% to 6 wt.%, all the microspheres were of high sphericity and integrity, and as expected, both *D*_mean_ and CV decreased with the increase in Span 80 content. When the Span 80 content further increased to 8 wt.% (sample F5) and 10 wt.% (sample F6), the values of *D*_mean_ and *D*_intra_ fluctuated slightly, but CV increased to 37.5% and 41.0%, respectively. These results suggested that excessive Span 80 caused the dispersity of TMPSQ microspheres to widen.

## 3. Materials and Methods

### 3.1. Materials

MTMS (AR) and MPTMS (AR) were obtained from 3A Chemicals Scientific Co., Ltd. (Shanghai, China). MeOH (AR) was supplied by Beijing Tongguang Fine Chemicals Inc. (Beijing, China). Hydrochloric acid (HCl, 35–37 wt.%, AR) was purchased from Modern Oriental Fine Chemistry Co., Ltd. (Beijing, China). Ammonium hydroxide (NH_4_OH, 25–28 wt.%, AR), ethanol (AR), and cyclohexane (AR) were obtained from Shanghai Titan Scientific Co., Ltd. (Shanghai, China). Liquid paraffin (AR, 0.83–0.86 g/mL of density, ≥300 °C of distillation temperature at atmospheric pressure) was purchased from Sinopharm Group Chemical Reagent Co., Ltd. (Shanghai, China). Span 80 (CP) was obtained from Shanghai Macklin Biochemical Technology Co., Ltd. (Shanghai, China). Water was deionized using a RO DI digital water purification system (Shanghai, China).

### 3.2. Preparation of TMPSQ Microspheres

The TMPSQ microspheres were prepared according to our previous report [25].

First, certain amounts of Span 80 and liquid paraffin were charged into a three-necked 250 mL flask to form the oil continuous phase by stirring, and the total mass of the two components was fixed at 80 g.

Then, certain amounts of MTMS, MPTMS, MeOH, 0.01 M HCl (aq), and water (Water-A) were first added to a round-bottom 50 mL flask equipped with a magnetic stirrer. After stirring for 2 h, an alkaline catalyst solution prepared from NH_4_OH (aq, 10 wt.%) and water (Water-B) was added to form the sol–gel disperse phase.

Finally, the sol–gel disperse phase was dropwise charged into the oil continuous phase, and the system was mechanically stirred at 500 rpm for 16 h in a water bath at 25 °C. To obtain the TMPSQ microspheres, the product was collected by centrifugation, washed with cyclohexane twice and by ethanol twice, and dried at 40 °C for 2 d.

### 3.3. Characterization

The morphology and internal structure of the TMPSQ microspheres were observed by an ULTRA 55 scanning electron microscope (SEM, ZEISS, Jena, Germany), operating at a 15 kV accelerating voltage. To obtain the internal structure information, the TMPSQ microsphere samples were ground in advance. Before characterization, the samples were sputtered with platinum. The particle sizes and size distributions were measured via a software called Nano Measurer 1.2 (Fudan University, Shanghai, China) based on SEM photos. The numerical mean diameter (*D*_mean_) and standard deviation (*σ*) were calculated by measuring 200 microspheres. The coefficient of variation (CV), which is an index of the polydispersity of particles, was calculated as follows:

(1) $$\mathrm{CV}=\frac{\sigma }{D_{\mathrm{mean}}}\times 100\%$$

The macroporous structures of the TMPSQ microspheres were analyzed using an AutoPore V 9620 porosimeter (Micromeritics, Norcross, GA, USA), operating at pressures from 0.001 to 410 MPa. The samples were dried at 100 °C for 2 h in advance using a vacuum drying oven (DZF-6020, Shanghai Honghua Instruments, Shanghai, China). Through the mercury intrusion method, the pore size distributions were determined, and total pore volumes (*V*_pore_), total porosities (*P*_total_), and surface areas (*S*_MIP_) were quantified.

The minimal points in the middle of the bimodal pore size distribution curves were used to distinguish interparticle spaces and intraparticle macropores, and the maximal points of the peaks at smaller pore sizes were considered to represent the intraparticle pore sizes (*D*_intra_). Based on the cumulative intrusion volume at different pressures, the volume of interparticle spaces (*V*_inter_) and the volume of intraparticle pores (*V*_intra_) were determined. The total volume of the sample (*V*_sample_) was calculated by dividing *V*_pore_ by *P*_total_, and after subtraction of *V*_inter_ from *V*_sample_, the total volume of microspheres (*V*_microsphere_) was estimated using Equation (2):

(2) $$V_{\mathrm{microsphere}}{=V}_{\mathrm{sample}}-V_{\mathrm{inter}}=\frac{V_{\mathrm{pore}}}{P_{\mathrm{total}}}-V_{\mathrm{inter}}$$

Then, the intraparticle porosity (*P*_intra_) was obtained from Equation (3):

(3) $$P_{\mathrm{intra}}=\frac{V_{\mathrm{intra}}}{V_{\mathrm{microsphere}}}\;\times \;100\%$$

## 4. Conclusions

In summary, the macroporous TMPSQ microspheres with controllable diameter and pore size were successfully synthesized via a facile strategy that integrated inverse suspension polymerization, two-step sol–gel, and phase separation processes. The composition of the sol–gel disperse phase had remarkable effects on the morphology and pore size of the TMPSQ microspheres. Higher MR_H2O/Si_ and MR_MPTMS/MTMS_, and lower MR_MeOH/Si_ and MR_NH4OH/Si_ would cause larger pores but less sphericity and integrity. By varying the above four ratios, the pore size, specific surface area, total porosity, and intraparticle porosity could be adjusted from 40 nm to 3774 nm, from 1.3 m^2^/g to 71.3 m^2^/g, from 47.9% to 74.7%, and from 29.1% to 51.3%, respectively. The size and morphology of the TMPSQ microspheres were significantly impacted by WR_W/O_ and Span 80 content, and excessive WR_W/O_ or insufficient Span 80 content would result in the damage to the TMPSQ microspheres. We believe that this work will provide insights into the controllable synthesis and property improvement of new functional PSQ microspheres and accelerate the application of such microspheres in different fields.

## Figures and Tables

**Table 1:** Influence of the MR_H2O/Si_ on the properties of TMPSQ microspheres ^a^.

Sample	MR_H2O/Si_	Water-A ^b^	Water-B ^b^	*D*_mean_	CV	*S*_MIP_	*V*_pore_	*P*_total_	*D*_intra_	*V*_intra_	*P*_intra_
		(g)	(g)	(μm)	(%)	(m^2^/g)	(mL/g)	(%)	(nm)	(mL/g)	(%)
A1	7:1	2.59	2.35	11.4	36.5	52.8	1.49	61.0	40	0.39	29.1
A2	8:1	3.13	2.89	11.5	37.9	35.3	1.85	63.9	56	0.54	33.9
A3	8.5:1	3.40	3.16	11.4	33.2	19.4	2.00	66.7	183	0.55	35.2
A4	9:1	3.67	3.43	12.6	36.9	3.6	3.07	72.3	1315	0.96	45.0
A5	10:1	4.22	3.98	13.6	42.5	2.3	3.37	74.3	2509	0.94	44.8

^a^ Dosages of MTMS, MPTMS, MeOH, 0.01 M HCl (aq), NH_4_OH (aq, 10 wt.%), Span 80, and liquid paraffin were 6.81 g, 1.96 g, 7.69 g, 1.20 g, 1.60 g, 4.80 g, and 75.20 g, respectively; ^b^ Water-A and Water-B refer to the water added in the acid-catalyzed step and the base-catalyzed step, respectively.

**Table 2:** Influence of the MR_MeOH/Si_ on the properties of TMPSQ microspheres ^a^.

Sample	MR_MeOH/Si_	MeOH	*D*_mean_	CV	*S*_MIP_	*V*_pore_	*P*_total_	*D*_intra_	*V*_intra_	*P*_intra_
		(g)	(μm)	(%)	(m^2^/g)	(mL/g)	(%)	(nm)	(mL/g)	(%)
B1	0:1	0.00	8.1	44.4	13.6	0.70	47.9	–	–	–
B2	1:1	1.92	11.3	36.3	1.6	2.15	67.4	2490	0.73	41.2
B3	2:1	3.85	10.3	32.7	4.4	2.81	73.9	1321	0.80	44.6
B4	3:1	5.77	10.6	35.6	13.2	2.23	70.5	833	0.74	44.3
B5/A3	4:1	7.69	11.4	33.2	19.4	2.00	66.7	183	0.55	35.2
B6	5:1	9.61	12.2	39.1	39.6	1.88	64.6	77	0.61	37.3

^a^ Dosages of MTMS, MPTMS, Water-A, 0.01 M HCl (aq), Water-B, NH_4_OH (aq, 10 wt.%), Span 80, and liquid paraffin were 6.81 g, 1.96 g, 3.40 g, 1.20 g, 3.16 g, 1.60 g, 4.80 g, and 75.20 g, respectively.

**Table 3:** Influence of the MR_NH4OH/Si_ on the properties of TMPSQ microspheres ^a^.

Sample	MR_NH4OH/Si_	NH_4_OH (aq, 10 wt.%)	Water-B	*D*_mean_	CV	*S*_MIP_	*V*_pore_	*P*_total_	*D*_intra_	*V*_intra_	*P*_intra_
		(g)	(g)	(μm)	(%)	(m^2^/g)	(mL/g)	(%)	(nm)	(mL/g)	(%)
C1	4.7 × 10^−3^:1	0.80	3.88	8.1	59.4	3.8	2.39	73.0	2882	0.93	51.3
C2	7.0 × 10^−3^:1	1.20	3.52	9.6	39.5	8.6	1.57	60.3	1044	0.64	38.2
C3/B5/A3	9.4 × 10^−3^:1	1.60	3.16	11.4	33.2	19.4	2.00	66.7	183	0.55	35.2
C4	11.7 × 10^−3^:1	2.00	2.80	11.5	39.5	28.6	1.38	55.2	151	0.52	31.9
C5	14.1 × 10^−3^:1	2.40	2.44	11.9	38.3	71.3	1.80	62.6	95	0.50	31.6

^a^ Dosages of MTMS, MPTMS, MeOH, Water-A, 0.01 M HCl (aq), Span 80, and liquid paraffin were 6.81 g, 1.96 g, 7.69 g, 3.40 g, 1.20 g, 4.80 g, and 75.20 g, respectively.

**Table 4:** Influence of the MR_MPTMS/MTMS_ on the properties of TMPSQ microspheres ^a^.

Sample	MR_MPTMS/MTMS_	MTMS	MPTMS	*D*_mean_	CV	*S*_MIP_	*V*_pore_	*P*_total_	*D*_intra_	*V*_intra_	*P*_intra_
		(g)	(g)	(μm)	(%)	(m^2^/g)	(mL/g)	(%)	(nm)	(mL/g)	(%)
D1	0:1	8.17	0	9.1	37.7	70.8	2.13	62.6	151	0.75	37.0
D2/C3/B5/A3	1:5	6.81	1.96	11.4	33.2	19.4	2.00	66.7	183	0.55	35.2
D3	1:4	6.54	2.36	9.8	62.3	2.1	3.28	74.7	2485	0.79	41.5
D4	1:3	6.13	2.95	12.1	48.7	1.3	2.95	74.7	3774	0.53	34.5
D5	1:2	5.45	3.93	8.3	48.2	4.7	1.35	59.4	–	–	–

^a^ Dosages of MeOH, Water-A, 0.01 M HCl (aq), Water-B, NH_4_OH (aq, 10 wt.%), Span 80, and liquid paraffin were 7.69 g, 3.40 g, 1.20 g, 3.16 g, 1.60 g, 4.80 g, and 75.20 g, respectively.

**Table 5:** Influence of the WR_W/O_ on the properties of TMPSQ microspheres ^a^.

Sample	WR_W/O_	Precursors	*D*_mean_	CV	*S*_MIP_	*V*_pore_	*P*_total_	*D*_intra_	*V*_intra_	*P*_intra_
		(mol)	(μm)	(%)	(m^2^/g)	(mL/g)	(%)	(nm)	(mL/g)	(%)
E1	0.11:1	0.02	4.9	35.7	15.9	1.24	61.3	151	0.23	23.1
E2	0.22:1	0.04	8.4	33.7	13.3	1.44	58.6	227	0.46	31.2
E3/D2/C3/B5/A3	0.32:1	0.06	11.4	33.2	19.4	2.00	66.7	183	0.55	35.2
E4	0.43:1	0.08	14.4	56.0	2.8	2.92	78.0	1606	0.82	49.9
E5	0.54:1	0.10	7.9	52.5	2.2	2.45	71.7	2883	0.60	38.1

^a^ MR_H2O/Si_, MR_MeOH/Si_, MR_NH4OH/Si_, and MR_MPTMS/MTMS_ were 8.5:1, 4:1, 9.4 × 10^−3^:1 and 1:5, and dosages of Span 80 and liquid paraffin were 4.8 g and 75.2 g, respectively.

**Table 6:** Influence of the Span 80 content on the properties of TMPSQ microspheres ^a^.

Sample	Span 80 ^b^	Span 80	*D*_mean_	CV	*S*_MIP_	*V*_pore_	*P*_total_	*D*_intra_	*V*_intra_	*P*_intra_
	(wt.%)	(g)	(μm)	(%)	(m^2^/g)	(mL/g)	(%)	(nm)	(mL/g)	(%)
F1	1	0.80	12.6	61.5	2.5	3.13	79.3	2076	0.93	53.1
F2	2	1.60	17.3	36.4	4.1	2.73	77.0	1049	0.86	51.4
F3	4	3.20	13.1	35.5	9.3	2.08	69.8	434	0.70	44.0
F4/E3/D2/C3/B5/A3	6	4.80	11.4	33.2	19.4	2.00	66.7	183	0.55	35.2
F5	8	6.40	12.1	37.5	13.3	1.86	69.9	227	0.51	38.8
F6	10	8.00	12.0	41.0	19.3	1.96	69.6	183	0.56	39.4

^a^ MR_H2O/Si_, MR_MeOH/Si_, MR_NH4OH/Si_, and MR_MPTMS/MTMS_ were 8.5:1, 4:1, 9.4 × 10^−3^:1, and 1:5, respectively. The mass of oil continuous phase was fixed at 80 g; ^b^ mass content relative to the oil continuous phase.

## Data Availability

Data are contained within the article.

## Supplementary Materials

The following supporting information can be downloaded at: https://www.mdpi.com/article/10.3390/molecules29122841/s1, Figure S1: (a) FTIR and (b) Raman spectra of the TMPSQ microspheres; Figure S2: Solid-state ^29^Si NMR spectrum of the TMPSQ microspheres. References [8,25,31] are cited in the supplementary materials.
