# Supplementary material for: Micron-Sized Thiol-Functional Polysilsesquioxane Microspheres with Open and Interconnected Macropores: Effects of the System Composition on the Porous Structure and Particle Size of the Microspheres
Source: Molecules. 2024 Jun 14;29(12):2841. doi: 10.3390/molecules29122841 (PMC11206636; doi:10.3390/molecules29122841)
Supplement: Supplementary file 1 [file molecules-29-02841-s001.zip › molecules-3005794-supplementary.pdf]

Supplementary Information

Micron-Sized Thiol-Functional Polysilsesquioxane Microspheres with Open and Interconnected Macropores: Effects of the System Composition on the Porous Structure and Particle Size of the Microspheres

Lu Han, Zhenyu Nie, Rongsheng Gao, Chengyou Kan *

Key Laboratory of Advanced Materials of Ministry of Education, Department of Chemical Engineering, Tsinghua University, Beijing 100084, People's Republic of China

* Correspondence: kancy@tsinghua.edu.cn

Characterization of the chemical structure of the TMPSQ microspheres

The fourier-transform infrared (FTIR) spectrum was obtained via a Nicolet 560 spectrometer (Thermo Fisher Scientific, Waltham, MA, USA) using the KBr tablet method. Raman spectroscopy was carried out on an HR800 micro confocal Raman spectrometer (Horiba JobinYvon, Paris, France) with an excitation wavelength of 633 nm. The solid-state ^{29}Si NMR spectrum was acquired using an NMR spectrometer (Bruker Avance III, 600 MHz, Karlsruhe, Germany).

Figure S1 presents the FTIR and Raman spectra of TMPSQ microspheres, which had been reported in our previous research [25]. In the FTIR spectrum (Figure S1(a)), the stretching vibration bands of Si–C (782 cm^{-1}), the symmetrical deformation vibration band of CH_3 (1274 cm^{-1}), and the stretching vibration bands of C–H in CH_3 (2971 cm^{-1}) and CH_2 (2935 cm^{-1}) proved the presence of organic side groups. In the Raman spectrum (Figure S1(b)), the characteristic stretching vibration band of S–H at 2571 cm^{-1} was evidence of the existence of thiol groups. Besides, bands at 2964 cm^{-1} and 2905 cm^{-1} in the Raman spectrum were attributed to the antisymmetric stretching vibration and symmetric stretching vibration of C–H in CH_3, which also suggested the existence of methyl groups. The solid-state ^{29}Si NMR spectrum of TMPSQ microspheres is shown in Figure S2. There were only two obvious peaks at −56.7 ppm and −65.7 ppm belonging to T^2 (linear) and T^3 (fully condensed) species, respectively [8]. No peaks attributed to T^0 (monomer) or T^1 (dimer) species were found in Figure S2, suggesting that the extent of the condensation reaction and the degree of crosslinking of TMPSQ microspheres reached a very high level [31].

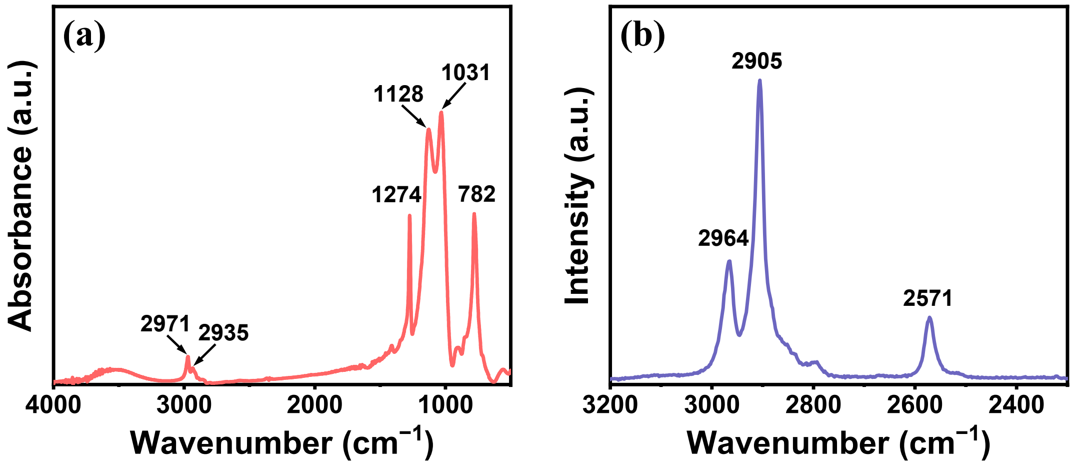

Figure S1. (a) FTIR and (b) Raman spectra of the TMPSQ microspheres [25].

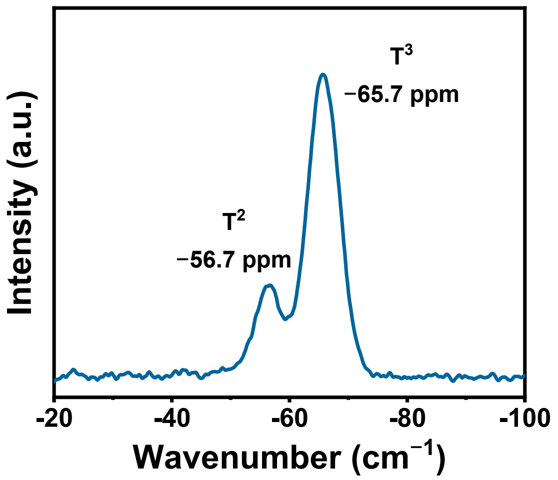

Figure S2. Solid-state ^{29}Si NMR spectrum of the TMPSQ microspheres.

References

8. Lu, X.; Hou, Y.H.; Zha, J.; Xin, Z. Size-controlled synthesis of monodispersed poly(3-mercaptopropylsilsesquioxane) microspheres by a two-step sol–gel method. *Ind. Eng. Chem. Res.* **2014**, *53*, 14659-14663. https://doi.org/10.1021/ie502146k.

25. Han, L.; Nie, Z.Y.; Gao, R.S.; Jiang, Z.Y.; Kan, C.Y. Micron-sized thiol-functional polysilsesquioxane microspheres with open and interconnected macropores: Preparation, characterization and formation mechanism. *Molecules* **2024**, *29*, 1204. https://doi.org/10.3390/molecules29061204.

31. Lu, X.; Hou, Y.; Zha, J.; Xin, Z. Facile synthesis of Rhodamine B-doped poly(3-mercaptopropylsilsesquioxane) fluorescent microspheres with controllable size. *Ind. Eng. Chem. Res.* **2013**, *52*, 5880-5886. https://doi.org/10.1021/ie302556t.
